# Remedy for Cough

**Published:** 1882-07

**Authors:** 


### REMEDY FOR A COUGH.

"THIS is the season for coughs and colds, and amid the changes of the weather too much care and caution cannot be taken in regard to them. There is a simple remedy for a cough, which we have occasionally tried, found effective, and can, therefore, recommend it. It is a mixture of one-third antimonial wine, one-third of syrup of squills, and one-third paregoric, in equal proportions, mixed.

"A swallow of it occasionally, so as to moisten the throat. It is innocent in its effects, and can, at any rate, do no harm.

"The apothecaries will sell a sixpence worth; but the best way for those who can afford it is to get it at some reliable druggist's—say twenty-five cents' worth of each in separate bottles, and mix it as they want it, themselves.

"In respect to the antimonial wine, an article is sometimes sold, we have reason to believe, where alcohol, instead of wine, is used in the preparation. In that case it would be too strong. Almost every druggist, of good reputation, however, has the preparation as it should be from its name. The only inconvenience from using the other, as already stated, is its being stronger than most persons can bear, and is too heating for the system.

"Persons having a cough, too, should be careful to abstain from the use of cold water as a drink, and also, as far as proper, from its use as an external application. A free bathing from pure alcohol, instead of water, of the throat and chest, to be wiped dry afterwards, would be of service.

"Persons having a cough, also, would find much benefit from wearing over the throat and chest a layer of cotton-flannel. It could be suspended by a string from the neck, or fastened inside of the garments. The cotton, of course, must be placed next to the flesh.

We need not say that care should at all times be taken to keep the feet dry and comfortable, and avoid, as far as possible, at any time, letting the blood in any way become chilled. Those most subject to coughs and colds are the poor, who have to expose themselves to the weather; and to such, and others, an observance of the above hints may not be unattended with at least some beneficial effects."

The above *remedy for a cough*, has been taken from a recent number of a religious newspaper, published in New-York, and we take occasion here to administer to this otherwise excellent periodical, in common with others of its class—a rebuke, which we hope will be felt and heeded. We cannot understand why it is, that the so-called *religious press* of this country is so steadily degenerating to secularization. With their shameful and crabbed abuse of each other—their vindictive denunciation of those who have another faith than their own—their spiteful, impatient and sweeping epithets of one another—the long columns of foreign correspondence, which encumber their pages, oftentimes having the very slightest religious bearing—the selling their advertising columns to the most bare-faced impositions on the credulity of their patrons—we say, with all these things before us, we are inclined to the belief, that without a more distinct line of demarkation between them and common newspapers, the sooner they are discontinued the better. With very few exceptions, there is not a religious newspaper in the United States whose editorials, in the course of one year, do not forfeit the title of a "*Christian gentleman.*" If the scripture rule be applied to them strictly, "*with what measure ye mete, it shall be measured to you again,*" very few of them, indeed, will have the invitation given—"*come sit ye up higher.*"

Ignorance is always reckless. It is the unpardonable recklessness of truth in reference to health, expressed in the article above referred to, which has, in this instance, provoked us to say hard things. It is within a month that we saw announced in a religious newspaper, published in the South, that during the year eighteen hundred and there was a very marked decline in deaths from consumption, and that it was to be attributed to the efficacy of "*Ayres' Cherry Pectoral.*" In another part of the same paper we noticed a long advertisement of this same medicine. In view of this, we simply inquire, what amount of respect is due any editor or proprietor, who will suffer himself to be bought over to such a palpable prostitution of his influence. But we wish more particularly to expose the shameful falsities contained in this vaunted *remedy for a cough.* It is given with the advantages of editorial experience, as it is not marked as quoted from any paper. One-third of the mixture is *Paregoric.* We think that editor would not have dared to recommend a cough remedy which was compose of over one-third brandy. We need enter into no argument to prove that opium intemperance is more terrible than that from brandy: and yet this editor declares it is innocent in its effects, and can, at any rate, do no harm. Is this true? *Antimonial wine* is alcohol, with a little tartar emetic in it; and *Paregoric* is alcohol, with a little opium in it. Of the bulk of this *remedy for cough*, two-thirds is alcohol, and one-third water. We believe regular topers think that a glass of *half-and-half* is pretty strong, and they are unanimous in the opinion that *it does no harm*—in fact, they say it does them good; but here is a religious editor, who declares, in effect, that an alcoholic mixture, with only one-third water, a swallow of it occasionally, "*is innocent in its effects, and can at any rate do no harm.*"

The writer goes on to say, that while it is innocent, and can do no harm to swallow opium, dissolved in alcohol, for a cough, it *does* do harm to drink cold water, and that instead of washing in cold water, it is better to wash with alcohol—that is to say, an "innocent" remedy for a cough, is to soak the inside with alcohol and opium, and the outside with alcohol alone, but water must be studiously avoided. The writer concludes in the following benevolent strain:—as the poor are more subject to coughs and colds, they should purchase fifty cents worth of alcohol, with a little tartar emetic and opium in it, and twenty-five cents worth of water, with a little squills, and take a swallow of this occasionally. Shame on the ignorance and inconsistency or negligence which could admit such an article in a paper, designed for religious family reading. And as to saying that opium, in any form, is innocent, or curative of any disease under the sun, none but a most unmitigated ignoramus would father such a sentiment.

